# Running to Stand Still: Naive CD8^+^ T Cells Actively Maintain a Program of Quiescence

**DOI:** 10.3390/ijms21249773

**Published:** 2020-12-21

**Authors:** Taylah J. Bennett, Vibha A. V. Udupa, Stephen J. Turner

**Affiliations:** 1 Department of Microbiology, Biomedicine Discovery Institute, Monash University, Clayton, VIC 3800, Australia; taylah.bennett1@monash.edu (T.J.B.); Vibha.Udupa@monash.edu (V.A.V.U.); 2 Department of Microbiology and Immunology, The Doherty Institute at the University of Melbourne, Parkville, VIC 3010, Australia

**Keywords:** CD8^+^ T cell, naïve T cell, memory T cell, transcription factors, epigenetics

## Abstract

CD8^+^ T cells play a pivotal role in clearing intracellular pathogens and combatting tumours. Upon infection, naïve CD8^+^ T cells differentiate into effector and memory cells, and this program is underscored by large-scale and coordinated changes in the chromatin architecture and gene expression. Importantly, recent evidence demonstrates that the epigenetic mechanisms that regulate the capacity for rapid effector function of memory T cells are shared by innate immune cells such as natural killer (NK) cells. Thus, it appears that the crucial difference between innate and adaptive immunity is the presence of the naïve state. This important distinction raises an intriguing new hypothesis, that the naïve state was evolutionary installed to restrain a default program of effector and memory differentiation in response to antigen recognition. We argue that the hallmark of adaptive T immunity is therefore the naïve program, which actively maintains CD8^+^ T cell quiescence until receipt of appropriate activation signals. In this review, we examine the mechanistic control of naïve CD8^+^ T cell quiescence and summarise the multiple levels of restraint imposed in naïve cells in to limit spontaneous and inappropriate activation. This includes epigenetic mechanisms and transcription factor (TF) regulation of gene expression, in addition to novel inhibitory receptors, abundance of RNA, and protein degradation.

## 1. Introduction

CD8^+^ T lymphocytes (CTLs) are the assassins of the adaptive immune system. They are crucial for inducing apoptosis of virally infected and cancerous cells, thus contributing to pathogen clearance and tumour elimination. Successful activation of naive CD8^+^ T cells requires presentation of pathogen-derived or tumour peptides by professional antigen-presenting cells (APCs) in the context of major histocompatibility molecule class I (MHC) molecules to the T cell receptor (TCR), in addition to receipt of co-stimulatory and inflammatory signals [1,2,3]. Activation of naive CD8^+^ T cells induces a program of proliferation and differentiation, resulting in the generation of an army of effector CD8^+^ T cells now able to secrete effector molecules such as the cytokines interleukin-2 (IL-2), tumour necrosis factor (TNF), and interferon-gamma (IFN-γ), as well as granzymes and perforins (cytotoxic molecules) [4,5,6]. Upon clearance of infection, the majority of antigen-specific CD8^+^ T cells die, while a small population persists and establishes long-lived memory [7]. Indeed, a cardinal feature of T cell-mediated immunity is establishment of immunological memory, where memory CD8^+^ T cells are capable of rapidly responding to re-infection, thus preventing disease [8]. The importance of CD8^+^ T cell-mediated immunity was highlighted in the 2009 H1N1 influenza A virus pandemic, where it was observed that people who had pre-existing antigen-specific CD8^+^ T cell immunity had reduced disease severity or were resistant to infection [9]. Recently, in response to the current global pandemic, human T cell responses to severe acute respiratory syndrome coronavirus 2 (SARS-COV2), the cause of Coronavirus Disease 2019 (COVID-19) have begun to be characterised by various groups. In Sweden, it was reported that robust CD8^+^ T cell memory is formed following infection [10]. Additionally, T cell cross reactivity between seasonal human coronaviruses and SARS-COV2 has been reported [11]. Thus, pre-existing T cell immunity may confer protection for people infected with SARS-COV2 and might explain why some people do not have severe disease. However, these investigations are ongoing. Furthermore, reactivating CD8^+^ T cell immunity against tumours using targeted checkpoint blockade therapy has revolutionised cancer treatments and patient outcomes, subsequently leading to the Nobel Prize in Physiology and Medicine in 2018 being awarded to James Allison and Tasuku Honjo for their discovery of CTLA-4 (cytotoxic lymphocyte protein 4) and PD-1 (programmed cell death protein 1) [12,13].

Dysregulated and inappropriate CD8^+^ T cell activation can underpin pathology in autoimmune diseases such as type 1 diabetes (T1D) and systemic lupus erythematosus (SLE) [14]. Spontaneous activation and differentiation are kept in check by mechanisms to enforce tolerance. One such mechanism is the requirement for naïve CD8^+^ T cells to overcome a threshold for activation. This is achieved upon receipt of sufficient strength and quality of activation signals, such as the requirement for co-stimulation and inflammation in addition to TCR signalling [3,15,16]. Therefore, understanding the mechanisms that uphold a barrier against activation and thus maintain the naive state are clearly paramount because inappropriate removal of this restraint results in the “killer” potency of CD8^+^ T cell activation and effector functions being unleashed and directed towards self-tissues. In the past, the quiescence of naive T cells has been described as a “default” or “resting” state (previously reviewed [17]). However, it is now clear that maintaining CD8^+^ T cell naivety is in fact an active process, one that we speculate has developed with evolution of the adaptive immune system to put a barrier on a default program of effector and memory differentiation, which is detrimental to the host under homeostatic conditions. Indeed, effector and memory CD8^+^ T cells have functional characteristics typically ascribed to innate immunity (rapid effector function upon activation). Hence, a crucial difference between adaptive and innate immunity is the presence of a true “naïve state”. In this review, we examine the restraint enforced in naive CD8^+^ T cells to limit immediate effector function, which is distinct from the “rapid fire” capacity of innate immune cells. This active process is one that receives input from multiple levels of regulation including epigenetic mechanisms, in addition to direct signals via inhibitory receptors, RNA metabolism, regulation of protein synthesis and degradation, and TF regulation. Here, we discuss these various inputs, which collectively set a threshold for the activation of naïve CD8^+^ T cells. This regulatory threshold must be overcome by the strength and quality of opposing activation signals.

## 2. Chromatin Landscape Forms a Molecular Barrier Limiting Naive T Cell Activation

Epigenetic mechanisms are considered key regulators of cellular differentiation. Major epigenetic mechanisms include covalent modifications of DNA and post translational modifications (PTMs) of histone proteins positioned within regulatory genomic elements such as promoters and transcriptional enhancers (TEs). DNA is coiled around a complex of histone proteins H2A, H2B, H3, and H4 forming an octamer. Histone proteins can undergo several types of PTM, including methylation, acetylation, and ubiquitination [18,19]. These modifications contribute to chromatin compaction or decompaction, subsequently regulating chromatin accessibility and gene transcription and/or acting as a platform for recognition and association with chromatin-modifying proteins. TEs can be positioned upstream, downstream (often hundreds of kilobases), or even within target genes that, once brought into close proximity to a promoter via chromatin folding, contribute to gene transcription [20]. TEs are often occupied by specific transcription factors, with chromatin looping bringing the TFs bound to enhancers into close proximity with a target gene promoter. Like gene promoters, histones associated with TEs can exhibit distinct PTMs that result in regulation of TE activity [21].

Methylation of DNA on cytosine residues within CpG dinucleotides is a stable modification that can be inherited after cell division. DNA methylation is mediated by DNA methyltransferases such as DNA methyltransferase 3a (DNMT3a) [22], while active removal of methylation is mediated by ten-eleven translocation (TET) enzymes [23]. DNA methylation represses transcriptional activation by acting to sterically hinder binding of transcription factors to target regulatory elements. Transcriptional regulation by DNA methylation and histone PTMs often intersect. For example, maintenance of DNA methylation status relies on H3 lysine 9 (H3K9) methylation by ensuring stable binding of DNMT enzymes to target CpG islands [24].

Modulation of permissive or repressive histone modifications have been shown to play a pivotal role in CD8^+^ T cell differentiation and acquisition of effector functions [25,26,27]. Similarly, DNA methylation-mediated transcriptional repression plays a key role in CD8^+^ T cell differentiation and function [28,29,30]. Together, DNA and histone modification within the promoter and enhancer regions provides a key epigenetic mechanism that is required for optimal effector differentiation. Naïve CD8^+^ T cells have a unique chromatin architecture and chromatin landscape that allows for the maintenance of naïve transcriptional programs, with it appearing that chromatin modifications in naïve cells provide a mechanistic basis for maintaining quiescence [26]. Moreover, the chromatin landscape is pre-configured in a way that enables rapid differentiation and proliferation upon activation to generate effector or memory cells. Here, we further discuss how regulation of chromatin in naïve CD8^+^ T cells plays a role in maintaining the quiescent state.

## 3. The Naïve Transcriptional Program Is Maintained by Permissive Chromatin Modifications and Is Shut Down upon Activation

CD8^+^ T cell differentiation states are underscored by expression of unique TFs, effector genes, and markers of effector/memory lineage commitment [31,32,33]. Naïve CD8^+^ T cells are characterised by a specific transcriptional profile and key markers of self-renewal and quiescence that maintain the stemness of the state, which are interestingly repressed during CD8^+^ T cell activation [32,34]. This includes special AT-rich binding protein (SATB1), main downstream effectors of the Wnt pathway such as T cell factor 1 (TCF-1, encoded by *Tcf7*), and lymphoid enhancer-binding factor 1 (LEF1 encoded by *Lef1*). SATB1 is a chromatin organizer capable of activating or repressing gene transcription [35]. SATB1 was described to regulate the coordinated expression of cytokines in T-helper 2 (Th2) cells by forming loops around 200-kb Th2 cytokine locus [36]. SATB1 has been shown to restrain PD-1 expression during CD8^+^ T cell activation by recruiting the nucleosome remodelling deacetylase complex to *Pdcd1* enhancer region [37]. SATB1 is strongly expressed in naive CD8^+^ T cells (in both mouse and human) and is repressed upon activation [26,38]. Thus, the actions of SATB1 in regulating the chromatin architecture to control gene transcription likely serves as a key epigenetic mechanism by which CD8^+^ T cell naivety is actively enforced.

Active shutdown of the naïve program during CD8^+^ T cell activation is required for effector differentiation and is associated with transition of the chromatin architecture from a permissive to repressive state at key TFs required to maintain the naïve state [26,33]. TCF1 is a key factor that ensures naïve and memory T cell self-renewal capacity [39]. T cell activation results in TCF-1 downregulation and is associated with dynamic alterations in the chromatin landscape around the *Tcf7* promoter. DNA methyltransferase 3a (DNMT3a) is strongly upregulated after CD8^+^ cell activation and is responsible for de novo DNA methylation of the *Tcf7* promoter [28,40]. Genome-wide H3K4 and H3K27 trimethylation mapping by Crompton et al. revealed that both the promoter and gene body of *Tcf7* was marked with high levels of H3K4me3, enforcing its constitutive expression in naïve cells [41]. Similarly, activating histone marks were observed at the promoter region of *Lef1.* The Forkhead O transcription factor 1 (*Foxo1*), a memory-associated gene, was also shown to be enriched with H3K4me3 in naïve CD8^+^ T cells [41]. This permissive signature associate with maintaining self-renewal capacity also extends to associated regulatory elements for naïve associated gene loci. Chromatin Immunoprecipitation-sequencing (ChIP-seq) analysis showed that putative TEs identified for *Tcf7, Lef1, Ccr7, Runx1, Runx2,* and *Satb1* were active in the naïve state but were decommissioned upon effector CD8+ T cell differentiation [42]. Taken together, these reports demonstrate that naïve CD8^+^ T cells actively maintain permissive chromatin around the genes that maintain the identity and stemness of naïve CD8^+^ T cells. Specific effector genes, such as *Tnfa, Ccl3, Xcl1*, and *Il2ra*, exhibit rapid transcription after naïve T cell activation. The *Tnfa* locus was shown to have an established permissive chromatin structure within naïve CD8^+^ T cells, consistent with an ability to be rapidly expressed after stimulation [25]. Other gene loci (such as *Ccl3, Xcl1,* and *Il2ra*) that are rapidly transcribed exhibit a different chromatin signature that is a combination of both the repressive H3K27me3 and active (H3K4me2/H3K4me3) marks. This signature appears to poise these gene loci in naïve CD8^+^ T cells, ensuring that they are appropriately repressed in resting naïve CD8^+^ T cells. Upon receipt of sufficient activation signals, H3K27me3 is rapidly removed, ensuring appropriate expression of these effector molecules [26].

Naïve CD8^+^ T cells are metabolically quiescent, with basal nutrient uptake, minimal biosynthesis, and basal metabolic rate, wherein they uptake glucose and fatty acids, and generate ATP through the tricarboxylic acid (TCA) cycle in contrast to effector cells, which are majorly dependent on glycolysis for energy production (reviewed in [43,44]). Key glycolytic enzymes such as lactate dehydrogenase (*Ldha*), triose phosphate isomerase (*Tpi1*), and hexokinase2 (*hk2*) progressively gain H3K4me3 from naïve to effector differentiation, which correlates with their activated gene expression [41]. This progressive chromatin remodelling around metabolic genes in naïve cells enables maximised energy production towards proliferation and effector molecule production upon activation and formation of immunological memory [41,45]. These results indicate that active maintenance of the naïve state is associated with permissive chromatin landscapes at both gene promoters and key regulatory elements.

## 4. Repressive Chromatin Modifications in Naïve Cd8^+^ T Cells Actively Restrains Effector Differentiation

A key outcome of naïve T cell activation is the acquisition of lineage specific function with differentiation [25,46]. Not surprisingly, the acquisition of CD8^+^ T cell function is associated with dynamic changes in the chromatin landscape at key transcriptional factors and effector proteins [25,26,47]. The promoters of effector genes, such as *Zbtb32*, *Gzmb* (encoding the effector molecule granzyme B), and *Ifng,* are heavily methylated in naïve cells, with little or no transcription repressing their expression [28,48]. Similarly, the inhibitory receptor *Pdcd1* (encoding PD-1) is also methylated in the naïve state with removal of DNA methylation occurring with differentiation from the naïve to effector state [29]. The demethylation of DNA at effector loci is regulated by TET proteins [23]. TET2, a member of this family of methylcytosine dioxygenases, is specifically induced upon TCR signalling [49]. Thus, TET2 proteins are specifically induced upon activation and act to epigenetically activate lineage-specific programs that help underpin effector CD8^+^ T cell differentiation. Effector gene loci, such as *Ifng* and *Gzmb,* are heavily marked by the repressive H3K27me3 PTM and exhibit and inaccessible chromatin structure in naïve cells, which then resolves into a permissive chromatin landscape upon effector CD8+ cell differentiation and transcriptional upregulation [25,26,42]. Effector molecules such as *Ccl3*, *Ccl5*, *Xcl1,* and markers of effector cells such as *Klrg2*, *Il-2Ra*, and *Prdm1* also have a repressive chromatin landscape characterised by increased H3K27me3 deposition in naïve cells [26] that resolves upon differentiation. Taken together, this evidence clearly demonstrates that the effector genes in naïve CD8^+^ T cells are configured in a transcriptionally repressed state that requires extensive remodelling to become activated. This in part explains why the acquisition of lineage-specific function is linked to extended CD8^+^ T cell differentiation [25,46].

## 5. Effector Differentiation-Associated Transcription Factors Are Poised in Naïve Cells

As outlined earlier, naïve CD8^+^ T cells are quiescent and exhibit a multipotent state. A key question therefore is that given there is the need for extended differentiation for acquisition of lineage-specific function, what mechanisms then regulate the rapid metabolic and proliferative response observed upon T cell activation to drive effector T cell expansion? Bivalency is a state where both activating and repressive histone modifications are observed in the promoter regions of genes. Our group has demonstrated that the promoters of genes encoding key transcription factors necessary for effector differentiation such as *Tbx21, Irf4,* and *Eomes* are poised in naïve cells, meaning that they have both H3K4me3 and H3K27me3 deposition on their promoter region and lose the H3K27me3 mark within a few hours of activation [26]. In fact, genome-wide analysis demonstrated that loss of H3K27me3 at gene loci was the major epigenetic outcome of T cell activation. Importantly, not only was there evidence of H3K4me3 and at these gene loci, but genes exhibiting H3K27me3 loss were involved in fundamental cellular processes such as cellular metabolism, rapid cell cycling, DNA replication, and cellular proliferation. This pre-configuration of a transcriptionally poised chromatin landscape is also evident at enhancers that regulate CD8^+^ T cell effector differentiation-associated genes such as *Tbx21, Prdm, Irf8, Fasl, Il2ra,* and *Klrg1*. In this case, these TEs exhibited H3K27me3 marks along with H3K4me1/2 in the naïve state but acquired H3K27Ac upon T cell differentiation, resulting in TE activation [42]. Hence, this poising of the CD8^+^ T cell effector state extends to both CD8+ effector gene promoters and associated gene regulatory elements. Together, this demonstrates that genes key for initiating the proliferative response upon naïve T cell activation are in fact primed for rapid transcriptional activation without the need for de novo deposition of H3K4me3. In this way, H3K27me3 deposition acts as a molecular hand brake, ensuring that transcriptional activation only occurs once TCR signals are received, enabling rapid engagement of these cellular programs. In this way, extended differentiation that results in acquisition of lineage-specific function occurs after molecular engagement of the proliferative response.

**Figure 1:**
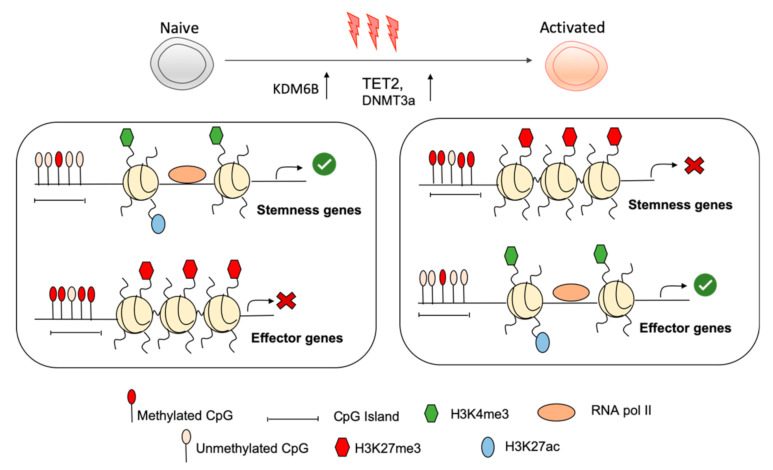
Epigenetic reprogramming within stemness and effector loci of naïve CD8^+^ T cells enables active maintenance of the naïve state and rapid shutdown of naïve programming during CD8^+^ T cell activation. Within naïve CD8^+^ T cells, stemness gene loci exhibit a permissive chromatin structure with deposition of H3K4me3, H3K27ac, and unmethylated CpG island, which allows their transcription while key effector gene loci are repressed by repressive histone modification such as H3K27me3 and H3K9me3 and methylation of CpG islands. During activation, stemness genes are shut down by activity of DNA methyltransferase 3a (DNMT3a) and repressive histone modifications. Chromatin remodelling within effector loci permits their appropriate expression by permissive histone modification.

A key question is what regulates removal of H3K27me3 to initiate this program of cellular differentiation upon naïve CD8^+^ T cell activation. Removal of H3K27me3 is catalysed by KDM6A and KDM6B demethylases [50]. In mature CD4^+^ T cells, KDM6A activity was required for the rapid expression of several key transcription factors, such as T-BET and STAT family members [51]. *Kdm6b*-deficient CD4^+^ T cells demonstrate dysregulated and inappropriate fate specification under T_H_ skewing conditions with promotion of T_H_2/T_H_17 lineages at the expense of T_H_1 and FOXP3 T regulatory cells [52]. We have demonstrated that KDM6B, but not KDM6A, is rapidly induced upon naïve CD8^+^ T cell activation [26]. We are currently examining the role of KDM6B in targeting H3K27me3 removal upon T cell activation and whether this results in the rapid engagement of the effector CD8^+^ T cell program (Figure 1).

## 6. Shutting Down the Naïve T Cell Program

With activation of an effector CD8^+^ T cell program, there is also the need to concurrently shut down the active naïve CD8^+^ T cell program. Polycomb repressive complexes (PRC) are chromatin remodelling complexes implicated in transcriptional repression [53]. PRC2, which catalyses H3K27me3, and PRC1, which mediates monoubiquitination of H2AK119, repress developmental regulators and lineage-specific genes in mouse embryonic stem cells (ESCs) and hence act to restrain cellular differentiation [54,55]. SUV39h is a histone methyltransferase responsible for trimethylation of H3 lysine 9 (H3K9) and is generally enriched in heterochromatin and it is involved in transcriptional silencing [56]. Once CD8^+^ cells are activated, epigenetic modulators such as SUV39h and PRC2 are induced. These epigenetic modulators shut down naïve transcriptional program by repressing and regulating effector/memory differentiation. SUV39h1 silences memory and stem cell programs during the terminal differentiation of effector CD8^+^ T cells [57]. In naive CD4^+^ T cells, EZH2, the enzymatic component of PRC2, has been shown to repress lineage specific cytokines in vitro by depositing H3K27me3 at target gene promoters and regulatory elements [58]. Enhancer of zeste homologue 2(EZH2) deletion in peripheral T cells was associated with increased production of IFN-γ and IL-10, indicating the inhibitory role of PRC2 in CD4^+^ T cell differentiation [59]. Taken together, these data show that T cell activation induces competing epigenetic mechanisms that serve to shut down the naïve T cell program (PRC2, SUV39H), whilst simultaneously acting to engage the necessary proliferative and differentiation programs (loss of H3K27me3, deposition of H3K4me3) that results in acquisition of lineage-specific CD8^+^ T cell function. A key question to ask is how competing mechanisms are specifically targeted to distinct gene promoters and regulatory elements. Moreover, what is unclear is what happens if there is incomplete shutdown of the naïve program. It is tempting to speculate that this may in fact lead to establishment of memory T cells, a differentiation state that exhibits hallmarks of both the naïve and effector state. Nevertheless, these observations all point to the fact that there is active maintenance of the naïve state via both the expression of stem cell-like gene programs, and restraint of the effector program. However, this restraint is on a knife’s edge, requiring T cell activation to rapidly unleash programmed CD8^+^ T cell proliferation and differentiation.

## 7. Engagement of the Effector CD8^+^ T Cell Program: Driven by Specific TFs

The naive state, enforced in part by the regulatory actions of a collection of key TFs as discussed, are opposed by other TFs that drive activation and subsequent effector differentiation. The upregulation of this specific set of TFs are important for driving effector differentiation and for maturation of CD8^+^ T cell responses. However, the induction of these TFs occurs only once T cells overcome a signalling threshold, which ensures that cell differentiation is only induced appropriately in response to infection. Interferon Regulatory Factor 4 (IRF4) is essential for driving effector differentiation, as genetic deletion of *Irf4* results in a failure of the expansion phase in the CD8^+^ T cell response to viral infection [60]. In fact, IRF4 has been shown to be a crucial regulator of the metabolic reprogramming that is required to enable clonal expansion by directly binding and activating genes involved in aerobic glycolysis [60]. IRF4 is induced rapidly (within hours) of activation, and in a TCR signal strength-dependent manner. High levels of IRF4 expression requires that T cells are induced with strong TCR signals that overcome the activation threshold, while weaker TCR signals that are unable to overcome the activation threshold do not induce strong IRF4 expression [60]. Thus, IRF4 induction is a molecular rheostat that is directly linked to the degree of TCR signal strength and subsequent engagement of T cell proliferative and differentiation programs.

IRF4 forms a complex with Basic Leucine Zipper Transcriptional Factor ATF-like (BATF), which is also a TF required for CD8^+^ T cell expansion and effector differentiation [61,62]. Together, BATF and IRF4 bind to gene promoters, resulting in transcriptional activation of genes encoding key TFs that regulate effector differentiation such as T-BET (encoded by *Tbx21*) and Blimp-1 (encoded by *Prdm1*), and genes encoding cytokine receptors such as the interleukin-12 (IL-12) and IL-2 receptors [62]. Simultaneously, the BATF–IRF4 complex represses the expression of genes encoding effector molecules that are downstream of these TFs and cytokine signalling pathways, such as IFN-γ and granzyme B [62]. Thus, such regulation by BATF and IRF4 ensures that a crucial checkpoint is met before the downstream “molecular handbrake” on effector molecule expression is released and full acquisition of the effector phenotype is permitted. T-BET is induced by TCR signalling and is crucial for generating effector CD8^+^ T cell responses through its direct activation of IFN-γ expression [63]. Indeed, mice lacking T-BET cannot generate effector CD8^+^ T cells equipped with potent cytotoxic functions [63,64]. Further, IFN-γ signalling itself drives T-bet expression to establish a feedforward loop that stabilises the effector CD8^+^ T cell differentiation state. Importantly, the presence of IL-12 strongly increases the amount of T-bet in a dose-dependent manner, which further drives the effector differentiation program [65], reinforcing the idea that a culmination of signal strength and quality is required to overcome the barrier set by negative regulatory signals.

## 8. Regulation by Transcription Factors Restrains Activation in Naïve T Cells

**Figure 2:**
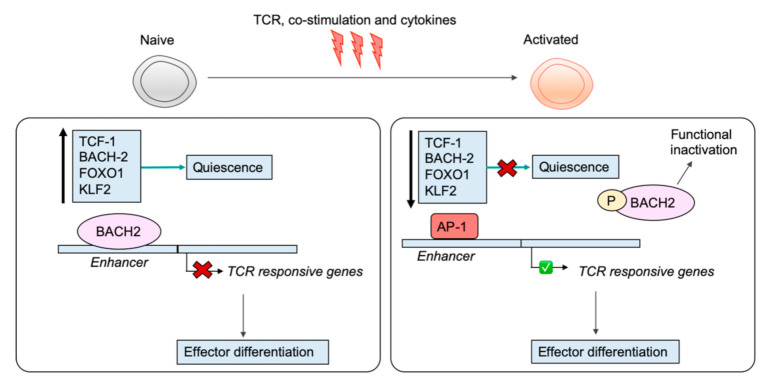
Key transcription factors enforce quiescence of naïve T cells. The transcription factors TCF-1, BACH2, FOXO1, and KLF2 are strongly expressed in naïve CD8^+^ T cells, where they exert crucial regulatory functions to uphold the naïve state. BACH2, for example, binds to enhancers, limiting the availability of AP-1-binding sites and repressing the expression of T cell receptor (TCR)-responsive genes. Upon activation, BACH2 is phosphorylated, leading to its functional inactivation, as well as being transcriptionally repressed. Removal of BACH2 releases the molecular brake on TCR-driven effector programs.

The different phenotypic and functional capacities of naïve, effector, and memory CD8^+^ T cells are underscored by unique transcriptional profiles that arise through the regulatory activities of differentiation state-specific TFs (reviewed Kaech et al. [34] and Russ et al. [32]). This is no less important in naive T cells, where a collection of key TFs have been documented to be crucial regulators for maintaining the naïve state by repressing key genes for activation and effector differentiation or by activating the transcription of key markers of quiescence. As mentioned, these TFs often have a distinctive pattern of expression, being strongly expressed in naïve cells and repressed in activated or effector cells, indicating that silencing of their transcription is likely crucial for successful T cell activation. The TF TCF-1 is well appreciated for its role in thymic development as well as establishment of CD8^+^ T cell memory [66,67,68]. It is also crucial for maintaining T cell naivety and is strongly expressed in naive T cells and rapidly downregulated upon receipt of TCR, co-stimulatory, and inflammatory signalling, which enables subsequent effector differentiation [69]. The requirement for inflammation via signals such as IL-12 to silence *Tcf7* indicates integration of distinct signalling pathways is required to overcome the threshold for activation. In naive CD8^+^ T cells, BACH2 represses TCR-responsive genes that drive effector differentiation by binding directly to enhancers and inhibiting access of AP-1 factors [70]. Upon activation, BACH2 transcription is silenced and BACH2 protein is phosphorylated, resulting in its functional inactivation, thus removing the BACH2 restraint on TCR-driven programs [71] (Figure 2). FOXO1, another well-known TF for maintaining the quiescence of naive T cells is also rapidly phosphorylated and degraded upon antigen stimulation of naive CD8^+^ T cells [72]. FOXO1 enforces the quiescence of naive CD8^+^ T cells by activating the expression of genes involved in trafficking to regulate naive T cell homing. Additionally, FOXO1 is necessary for the survival of naïve T cells and activates the transcription of the IL-7R by binding to an IL-7R enhancer [72]. Loss of FOXO1 disrupts homeostasis of the naive T cell compartment, as demonstrated by an accumulation of CD44 high CD4^+^ and CD8^+^ T cells [72]. FOXO1 also induces Krüppel-like factor 2 (KLF2, also known as lung Krüppel-like factor 2) expression in naive T cells, another TF crucial for maintaining the naive state [73]. Indeed, naive T cells deficient for KLF2 are hyperproliferative and inappropriately express activation markers [74]. KLF2 enforces quiescence in naive T cells by repressing *Myc*, an important regulator of proliferation and metabolic reprogramming in activated T cells [73,75,76]. Taken together, the regulatory actions of TFs are crucial for maintaining the naïve program, and their removal upon activation is necessary for permitting acquisition of the effector phenotype (Figure 2).

## 9. At the Cell Surface: The Inhibitory Receptor VISTA Enforces T Cell Naivety

To ensure that effector T cell activation is tightly regulated, the role of inhibitory receptors in repressing T cell function during infection and cancer, particularly CTLA-4 and PD-1, is well appreciated. Engagement of these inhibitory receptors expressed on the surface of T cells represses T cell activation and functions to limit immunopathology in the host during infection, as well as preventing autoimmunity (reviewed by Andrews et al. [74]). However, tumours unfortunately target CTLA-4 and PD-1 to evade T cell-mediated immunity [74]. The development of checkpoint blockade therapy (treatment with antibodies to block ligand binding to CTLA-4 and PD-1 receptors) has indeed revolutionised cancer treatment and patient outcomes. A key distinction regarding these inhibitory molecules is that they are specifically expressed following T cell activation and therefore do not appear to play a role in maintaining the quiescent state of naïve T cells, despite their clear negative regulatory functions.

Key characteristics of naïve T cells is that they are small in size and “quiet” in terms of metabolic activity, energy utilisation, and turnover. This quiescence is often presented as a dormant state with little activity. However, there are in fact active processes required to maintain the naïve T cell state. As outlined above, epigenetic mechanisms appear to do this by providing a molecular barrier that must be overcome to result in both acquisition of lineage specific function, as well as shutting down self-renewal and naïve T cell programs. Recently, there now appears a role for inhibitory receptors in actively enforcing this quiescence. The inhibitory receptor V-domain Ig suppressor of T cell activation (VISTA, also known as PD-1 homologue, PD-1H) was identified to be selectively expressed on naïve T cells as opposed to activated T cells, where it restrains activation [77]. Previous work has demonstrated that VISTA restrains T cell activation and proliferation in a co-inhibitory capacity via its expression on APCs, where it acts as a ligand to suppress T cell responses [78]. Disruption of this pathway impairs tolerance and leads to the development of autoimmunity [79,80,81]. More recently, ElTanbouly and colleagues demonstrated that intrinsic VISTA directly enforces T cell naivety via inhibitory signals [77]. By employing a combination of single cell RNA-sequencing and ATAC-sequencing approaches in addition to in vivo models, ElTanbouly et al. robustly interrogated the role of VISTA in the T cell compartment [77]. Loss of VISTA resulted in a reduction of the naive T cell population and accumulation of CD44 high memory phenotype cells [77]. In addition, the gene signature in VISTA-deficient T cells was similar to that of T cells derived from SLE and rheumatoid arthritis (RA) patients [77]. Thus, without the restraint imposed by VISTA, T cells enter a program of differentiation even under homeostatic conditions, indicating that VISTA upholds a barrier against inappropriate activation in naïve T cells, which might otherwise result in autoimmune disease. Furthermore, VISTA was required for the expression of multiple known regulators of the naive program, such as KLF2 and BTG1/2 [77]. VISTA potentially enforces T cell naivety in part by regulating the epigenetic program landscape at key gene loci, as loss of VISTA disrupts the quiescent program at the epigenetic level. This was characterised by increased chromatin accessibility at TCR-responsive genes and at gene loci that are typically associated with memory formation, although the precise mechanism by which VISTA regulates chromatin architecture is unclear [77]. Altogether, this reinforces the notion that naïve T cells are in fact epigenetically and transcriptionally poised to respond to TCR signals. In this context, VISTA serves to restrain inadvertent or inappropriate activation by enforcing the naïve T cell state, just as H3K27me3 deposition at transcriptionally poised (H3K4me3) loci. Importantly, VISTA expression on antigen-specific T cells was downregulated by inflammation, permitting T cell activation and differentiation [77], indicating that antigen stimulation in addition to inflammatory signals are required to overcome a threshold to silence the negative regulatory function of VISTA and subsequently achieve appropriate activation. Additionally, the requirement for inflammation to downregulate VISTA highlights the role of signal three and therefore signal “quality” in overcoming the regulatory threshold. Of note, this study examined the CD4^+^ T cell compartment and it remains to be confirmed whether these crucial observations apply to CD8^+^ T cells. Given that VISTA is expressed on CD8^+^ T cells under steady-state conditions [78], we predict that it likely functions in a similar capacity to also maintain the naive state of CD8^+^ T cells. Taken together, VISTA is an example of an inhibitory molecule that imposes active restraint of naïve T cell activation.

## 10. Regulation of RNA Degradation and Protein Abundance Are Crucial for Maintaining the Quiescence of Naïve T Cells

The above data suggests that naïve T cells actively maintain quiescence. As we have briefly described, naïve CD8^+^ T cells have a distinctive metabolic profile in comparison to effector CD8^+^ T cells [43,44]. This plays a role in enforcing quiescence and reflects the different requirement for energy utilization by naïve and effector T cells (recently reviewed comprehensively by Chapman and colleagues [82]). Key characteristics of the metabolic program in naïve CD8^+^ T cells are reduced glucose uptake and utilization of oxidative phosphorylation (OXPHOS), which is a highly efficient way for cells to produce ATP [83]. Upon activation, metabolic reprogramming occurs, and aerobic glycolysis is utilized rather than OXPHOS [83,84]. The mammalian target of rapamycin complex 1 (mTORC1) senses and integrates various signal inputs to promote glycolysis and drive proliferation [85]. The activity of mTORC1 is repressed in naïve T cells by the tuberous sclerosis complex 1 (TSC1) [86,87]. Loss of TSC1 results in activation of mTORC1 and disruption of T cell quiescence, indicating that regulation of metabolism plays a crucial role in maintaining the naïve program [86,87].

More recently, it has been elucidated that processes involved in regulating factors such as RNA metabolism and protein turnover may also be key mechanisms that maintain this state of “inactivity”. Hwang and colleagues identified that BTG1 and BTG2 (BTG1/2), which are members of the B cell translocation gene/transducer of ERBB family, are crucial for maintaining the quiescent state of naïve T cells [88]. BTG1/2 promotes the degradation of messenger RNA (mRNA), a previously unappreciated factor for maintaining the quiescent program [88]. Conditional deletion of BTG1/2 in T cells was detrimental to the naïve state, resulting in a reduced threshold for activation and hyperproliferation in response to stimulation [88]. Additionally, a reduction in the naive CD4^+^ and CD8^+^ T cell compartment and accumulation of effector memory (CD44^high^ CD62L^lo^) was observed in unchallenged mice [88], reminiscent of the phenotype of VISTA-deficient T cells and suggestive of inadvertent activation under homeostatic conditions [77]. This distinctive phenotype of spontaneous activation upon removal of BTG1/2 was explained by increased polyadenylate tail length and an associated increase in mRNA half-life, resulting in a build-up of mRNA molecules [88]. Much like other key regulators of naïve T cells such as TCF-1, BACH2, FOXO1, and KLF2, which we have previously discussed, BTG1/2 are strongly expressed in quiescent T cells and repressed upon activation [88]. The downregulation of BTG1/2 enables accumulation and enhanced stability of mRNAs transcribed upon T cell activation [88]. Thus, it appears that the quiescent state is indeed one where there is active transcription of key genes but in which restraint on activation is imposed in part because available mRNA is limited by BTG1/2. Complementing this study, Wolf et al. recently documented the dynamics of protein turnover in human naïve and activated CD4^+^ T cells [89]. In this powerful study, Wolf et al. combined heavy isotope-labelled amino acid incorporation to identify proteins that were newly synthesised with quantitative proteomics and transcriptomics to measure total copy numbers of proteins and mRNA molecules in naive versus in vitro-activated T cells [89]. Interestingly, naive T cells were documented to make ≈60,000 proteins per minute (in comparison to T cells activated for 24 h, which make ≈800,000 proteins per minute), but there was no overall change in protein abundance because there were rapid rates of protein degradation and synthesis [89]. The proteins that were constantly degraded and resynthesized were those that are well known to be crucial for enforcing the quiescent state of naive T cells such as the TF FOXO1, which had a half-life of 5 h [89]. The short half-life of these proteins means that with their transcriptional repression upon activation (a characteristic gene expression pattern for quiescent markers), the protein will be rapidly destroyed, permitting activation with the removal of this threshold. These data clearly demonstrate that naive T cells must actively and constitutively produce proteins that are essential to maintain the naive state.

## 11. Concluding Remarks

Cardinal features of adaptive T cell immunity are TCR recognition of cognate antigen and the establishment of long-lived immunological memory. A defining feature of innate immunity is rapid effector function upon receipt of specific activation signals. This characteristic is also one that is found in effector and memory CD8^+^ T cells within the adaptive immune system. Effector and memory CD8^+^ T cells share similar epigenetic profiles, characterised by permissive chromatin structures around key effector loci [25,70]. Thus, although quiescent, memory CD8^+^ T cells are epigenetically poised to immediately transcribe effector genes and engage effector functions following reinfection. In this respect, memory CD8^+^ T cells share functional characteristics of innate immune cells, which provide immediate responses to infection and can also form “memory”, engaging in recall responses. Indeed, memory CD8^+^ T cells share epigenetic profiles with both naive natural killer (NK) cells and “memory” NK cells [90]. Hence, at a mechanistic level, innate, effector, and memory immune cells exhibit functional similarities. The crucial and unique distinction then between innate and adaptive immunity is the restraint enforced in naive CD8^+^ T cells to limit immediate effector function, which is distinct from the “rapid fire” capacity of innate immune cells. We propose that with evolution, the adaptive immune system has likely installed molecular programming to enforce the “naive state” and thus impose restraint on effector and memory differentiation. Natural killer T (NKT) cells and mucosal-associated invariant T (MAIT) cells are innate like lymphocytes, which have a conventional TCR but exhibit innate-like characteristics such as immediate effector function, therefore bridging the innate and adaptive immune systems [91]. However, natural killer T (NKT) cells and mucosal-associated invariant T (MAIT) cells are limited in what activates them as they recognise non-peptide ligands presented on non-classical MHC molecules [92,93]. With the emergence of the evolutionarily advantageous antigen receptor diversity, diversification of classical MHC pathogen-derived targets can be recognised. Consequently, diversification of antigen presentation and recognition necessitated an adaption that effectively established functional and epigenetic barriers that limited naive T cell activation and avoided inappropriate or inadvertent activation. Thus, we speculate that the naive state was established. Here, we discussed that programs of quiescence in naïve T cells are actively maintained and establish a threshold that needs to be overcome for optimal CD8^+^ T cell activation. Dysregulation of these restraint programs results in lowering or removal of this activation threshold, and likely contributes to the development of autoimmunity or excessive antigen-specific responses to intracellular pathogens. Thus, in designing novel vaccine strategies to induce CD8^+^ T cell activation and subsequent responses that are robust, there should be consideration towards targeting the negative regulators that uphold the threshold for activation. Similarly, these factors that enforce this “molecular handbrake” in naive CD8^+^ T cells could represent potential targets of novel therapeutic strategies for the treatment of autoimmune disease in order to re-establish the quiescent program and limit disease. Targeting VISTA could perhaps represent one such strategy, as anti-VISTA agonists showed promise in the report by ElTanbouly et al. in inducing T cell tolerance [77]. Additionally, in these approaches, there should be consideration for targeting TFs that regulate quiescence at the transcriptional or epigenetic level. This might be more efficient than targeting protein degradation, as they have a short half-life and are rapidly resynthesized [89]. Ultimately, understanding the molecular programs that enforce the naive state will enhance our overall understanding of CD8^+^ T cell immunity, likely providing novel insight into more targeted interventions to restrain inflammation in the context of autoimmune disease or to improve cellular immunity against intracellular infections and tumours.

## Abbreviations

APC	Antigen-presenting cell
CTL	Cytotoxic T lymphocyte
IL-2	Interleukin-2
IFN-γ	Interferon gamma
MHC	Major histocompatibility complex
NK	Natural killer
PTM	Post-translational modification
TCR	T cell receptor
TE	Transcriptional enhancer
TF	Transcription factor
TNF	Tumour necrosis factor
